# Carbon Nanostructure-Based Field-Effect Transistors for Label-Free Chemical/Biological Sensors

**DOI:** 10.3390/s100505133

**Published:** 2010-05-25

**Authors:** PingAn Hu, Jia Zhang, Le Li, Zhenlong Wang, William O’Neill, Pedro Estrela

**Affiliations:** 1 Key Lab of Microsystem and Microstructure, Harbin Institute of Technology, Ministry of Education, No. 2 YiKuang Street, Harbin 150080, Heilongjiang, China; E-Mail: wangzl@hit.edu.cn (Z.W.); 2 Research Centre for Micro/Nanotechnology, Harbin Institute of Technology, No. 2 YiKuang Street, Harbin 150080, Heilongjiang, China; 3 Centre for Industrial Photonics, Institute for Manufacturing, Department of Engineering, University of Cambridge, 17 Charles Babbage Road, Cambridge, CB3 0FS, UK; E-Mail: wo207@cam.ac.uk; 4 Department of Electronic & Electrical Engineering, University of Bath, Bath, BA2 7AY, UK; E-Mail: p.estrela@bath.ac.uk

**Keywords:** chemical and biological sensors, carbon nanotubes, grapheme

## Abstract

Over the past decade, electrical detection of chemical and biological species using novel nanostructure-based devices has attracted significant attention for chemical, genomics, biomedical diagnostics, and drug discovery applications. The use of nanostructured devices in chemical/biological sensors in place of conventional sensing technologies has advantages of high sensitivity, low decreased energy consumption and potentially highly miniaturized integration. Owing to their particular structure, excellent electrical properties and high chemical stability, carbon nanotube and graphene based electrical devices have been widely developed for high performance label-free chemical/biological sensors. Here, we review the latest developments of carbon nanostructure-based transistor sensors in ultrasensitive detection of chemical/biological entities, such as poisonous gases, nucleic acids, proteins and cells.

## 1. Introduction

A chemical/biological sensor can be defined as a device that responds to changes in its chemical/biological environment and converts this response into a signal that can be read. As for the basic characteristics of a useful sensor, its response should be predictable so that it scales with the magnitude of changes in the chemical/biological environment, and the sensor should be sensitive and specific. In addition, the transduced signal can be electrical, magnetic, optical, *etc.* The development of chemical/biological sensors based on microtechnology is well established, as evidenced by an overwhelming number of published reports [1,2]. However, as nanotechnology has developed over the past twenty years, scientists have made significant efforts to develop sensor architectures with higher sensitivity, decreased energy consumption and miniaturized size. Huge progress in synthesis and our fundamental understanding of the physical and chemical properties of nanomaterials has stimulated interest in the incorporation of these novel materials into sensor architectures. Nanomaterials including quantum dots and one-dimensional nanostructures (e.g., nanowires, nanobelts and nanotubes) hold great promise for the development of miniaturized chemical and biological sensors. Their reduced dimensions generate novel physical properties and high surface to area ratio and thus lead to an increase in environmental sensitivity [3]. For example, optical semiconducting quantum dots can be effectively used for medical imaging. Carbon nanostructures, including carbon nanotubes (CNTs) and graphene, are composed almost entirely of surface atoms and have greater modulation of electrical properties (e.g., capacitance, resistance) upon exposure to analytes. Furthermore, their structural characteristics and electrical properties render them to be easily configured as field-effect transistors (FETs) and potentially integrated to form complex microelectronic systems with determined performance.

Carbon nanostructures including CNTs and graphene are central materials in nanoscience. Their unique electrical, physical, mechanical and chemical properties are widely studied so as to develop high performance devices. Thousands of related reports can be found in the relevant literature. This review is intended to discuss the latest progress in carbon nanotube and graphene based chemical/biological field-effect transistor sensors and present an outlook for the future of carbon nanotube and graphene based sensor technology.

## 2. Carbon Nanotube Chemical/Biological Sensors

### 2.1. Carbon Nanotubes and Carbon Nanotube Field-Effect Transistors

Since the discovery of CNTs in 1991 by S. Iijima [4], a great deal of effort has been devoted to the fundamental understanding of their electrical, mechanical and chemical properties and of their use in a wide range of applications such as electronics and sensors. CNTs are divided into single walled carbon nanotubes (SWNT) and multiwalled carbon nanotubes (MWNT). A SWNT can be formed by rolling a graphene sheet (hexagonal structure) into a cylinder and a MWNT is composed of concentric graphene cylinders with an interlayer spacing of 0.34 nm. Most of CNTs are synthesized by arc discharge, laser ablation or chemical vapor deposition method.

CNT properties are highly dependent on their structure. SWNTs can be either metallic or semiconducting, a property which is determined by the atomic arrangement (chirality) and nanotube diameter. The roll-up vectors (*n*,*m*) of the cylinder describe the electrical properties of SWNTs [5,6]. Metallic SWNTs have roll-up vectors such that *n* – *m* = 3*q* (where *q* is any integer or zero) and semiconducting SWNTs have *n* – *m* ≠ 3*q*. Semiconducting SWNTs are p-type semiconductors with holes as the main charge carriers. The bandgap energies of semiconducting SWNTs are inversely related to their diameters. MWNTs show metallic electronic properties similar to metallic SWNTs. Interestingly, because of the one-dimensional structure, MWNTs and metallic SWNTs behave ballistic electronic transport properties without scattering over long lengths. These properties allow CNTs to carry high currents with negligible heating [7].

Semiconducting SWNTs play a central role in the operation of SWNT-based field-effect transistors (SWNT-FETs). The first SWNT-FET was demonstrated in 1998 by the groups of Dekker *et al.* [8] and Avouris *et al.* [9]. SWNT-FET devices are composed of individual SWNTs or random networks of SWNTs placed between a source (S) and a drain (D) electrode on a SiO_2_/Si substrate. The Si layer can act as back gate, which is separated by an insulating layer of SiO_2_. Since the work function of SWNTs is higher than that of most metals, the contact barrier between SWNTs and metals is usually a Schottky barrier (SB). The height of the SB in the SWNT-FET contact is determined by the work function of the electrode metal [10]. The conductance of SWNTs in devices can be modulated by applying a potential to the gate electrodes with a constant D-S bias voltage (*V*_DS_).

### 2.2. Chemical CNT-FET Sensors

Sensing gas or organic vapor molecules is central to environmental monitoring, control of chemical processes, health protection and agricultural applications. For example, NO_2_, which can lead to respiratory symptoms in humans and dramatically affect agriculture, is produced from combustion or automotive emissions, and detection of NO_2_ is important to monitoring environmental pollution. SWNT-FET devices are ideal for monitoring environmental gases and organic vapors. SWNTs are entirely composed of surface atoms, which make their electrical properties sensitive to their chemical environment. Since semiconducting SWNTs are p-type under ambient conditions, electron-donating molecules such as NH_3_ that interact with SWNTs will result in charge-carrier (hole) recombination. This will cause a decrease in conductance and a shift in the transfer curve (*I*_DS_ – *V*_GS_) to more negative voltages. However, electron-withdrawing molecules such as NO_2_ and O_2_ can increase hole concentration in the SWNT-FET and thus increase conductance, which leads to a shift of the *I*_DS_ – *V*_GS_ transfer curve to more positive voltages.

So far, SWNT-FET sensors have been shown to be sensitive to gases such as NH_3_, NO_2_, H_2_, CH_4_, CO, H_2_S and some organic vapors such as ethanol and methanol. Firstly, the reported sensing devices are directly made from pristine CNTs. Subsequently, some gas sensor devices have been composed of functionalized CNTs, because gas sensors based on pristine CNTs have some shortcomings including low sensitivity to analytes due to low adsorption, low selectivity or long recovery time. To overcome the above shortcomings, many efforts have made to modify CNTs with different chemical materials to change their chemical nature and improve sensing performance.

**Figure 1.**
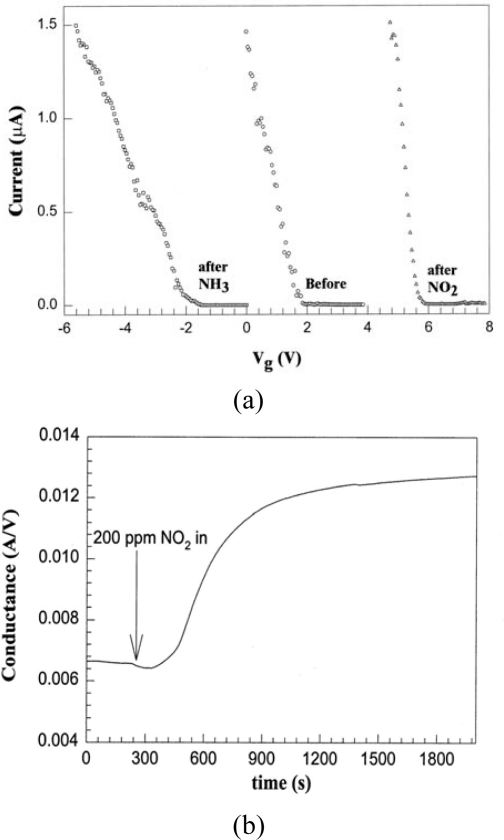

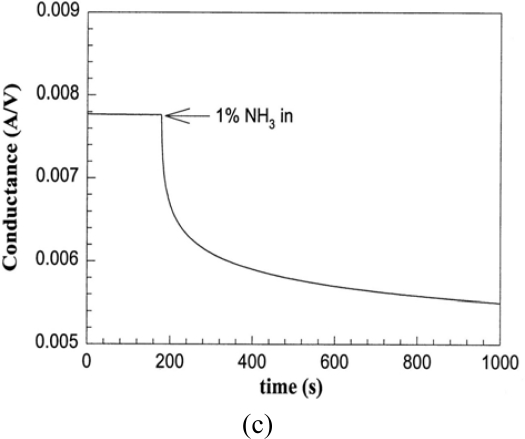
Chemical gating effects to the semiconducting SWNT. (a) Current *versus* gate voltage curves before NO_2_ (circles), after NO_2_ (triangles), and after NH_3_ (squares) exposure. (b) Time dependence of the conductance upon NO_2_ exposure. (c) Time dependence of the conductance upon NH_3_ exposure; Reproduced with permission from [11].

In 2000, Dai *et al.* first demonstrated a CNT-based gas sensor [11]. The FET consisting of individual semiconducting SWNTs was made by growing SWNTs via CVD on SiO_2_/Si substrates, then photolithographically patterning metal electrode on a single SWNT. The conductance of the SWNT-FET varied dramatically under various gate voltages when the device was exposed to NO_2_ or NH_3_ (shown in Figure 1). Upon exposure to NO_2_, the conductance of the FET increased by about three orders of magnitude, and the transfer characteristics showed a shift of +4 V in gate voltage. Conversely, when the FET was exposed to NH_3_ its conductance decreased by about three orders of magnitude and the transfer characteristics showed a shift of −4 V in gate voltage. The response time, defined as the time duration for signal stability from the introduction of sample, ranged from 2 to 10 s with 200 ppm of NO_2_. The sensitivity, defined as the ratio between resistance after and before gas exposure, was ∼100 to 1,000. The used FET sensor recovers its original properties slowly at room temperature, and a typical recovery time is about 12 h. However, heating exposal sample in air at 200 °C led to recovery in ∼1 hour. As for sensing NH_3,_ the response time to 1% NH_3_ was about 1 or 2 minutes, and sensitivity was from 10 to 100 ppm. Recently, Mattmann investigated CNT-FETs with aluminum oxide Al_2_O_3_ passivated contacts for NO_2_ detection with very low limit concentration (80 ppb). They found that preventing metal contact exposure to the environment (especially oxygen) could benefit the NO_2_ detection [12].

The sensing mechanism of NO_2_ was explained as an attraction of electron charge from the SWNTs. The theoretical result shows that an electron charge of 0.1 transfers from the SWNT to an adsorbed NO_2_ molecule. As for sensing NH_3_, the sensing mechanism remains unclear, since calculation find no binding affinity between NH_3_ molecules and SWNTs. The authors suggested that interaction between NH_3_ and SWNTs was through binding of NH_3_ to hydroxyl on a SiO_2_ substrate, which could partially neutralize the negatively charged groups and lead to a positive electrostatic gate to the transistor. Recently, Marzari *et al*. systematically studied sensing mechanisms for CNT-based NH_3_ sensors [13]. They found that the SB modulation at the CNT/metal contacts dominates the sensing performance at room temperature while at higher temperatures such as 150 °C or above, charge transfer process contributes to the sensing signal and NH_3_ adsorption is to be facilitated by environmental oxygen.

To improve the sensitivity, SWNTs in the device were non-covalently functionalized with organic polymers made by drop-coating or dip-coating methods. Qi *et al.* demonstrated that non-covalently drop-coating of polyethyleneimine (PEI) and Nafion (a polymeric perfluorinated sulfonic acid ionomer) onto SWNT-FETs resulted in gas sensors with improved sensitivity and selectivity for NO_2_ and NH_3_ [14]. The PEI functionalization changed the SWNTs from p-type to n-type semiconductors, and sensors were able to detect less than 1 ppb NO_2_ while being insensitive towards NH_3_. In contrast to PEI-coated sensors, Nafion-coated SWNTs were insensitive to NO_2_ while exhibiting a good sensitivity to NH_3_. Star *et al.* also fabricated non-covalently functionalized SWNT FETs by simply submerging nanotube network FETs in an aqueous solution of PEI [15].

Since this first report on SWNT-FET based gas sensor, a great deal of effort in development of CNT based gas sensor has been made for various gases such as H_2_, CH_4_, CO, H_2_S and some organic vapors. Since bare CNTs do not show obvious sensing for H_2_, work has been done in functionalizing CNTs with H_2_ or CH_4_ sensitive materials. Dai *et al.* first reported the sensitivity of Pd decorated SWNTs to ppm levels of H_2_ gas [16]. With the SWNT device coated with Pd nanoparticles by electron beam evaporation of Pd, the conductance of the Pd-modified device decreased upon exposure to H_2_. This H_2_ CNT sensor showed a response time of a few seconds and a recovery time of about 400 seconds. The sensing mechanism was explained as a dissociation of H_2_ into atomic hydrogen on the Pd surface, which decrease the Pd work function and causes the electron donation to SWNTs.

SO_2_ results from activities such as combustion and petroleum refining and can do harm to the environment since it can aid the formation of acid rain. SO_2_ can be adsorbed onto bare CNTs with an effect similar to NO_2_, but very few reports have been presented on CNT-based sensors for this gas. Suehiro *et al.* demonstrated that SWNT-FET shows a device behavior consistent with an electron donating species upon exposure to SO_2_ [17]. It remains unclear why SO_2_ can work as an electron donor in this procedure. H_2_S is a dangerous gas because of its flammability and toxicity. Star *et al.* reported a metal decorated SWNT sensor array for detecting 50 ppm H_2_S in air [15]. So far the reports of CNT-based sensors for the detection of SO_2_ and H_2_S are scarce, and further investigation into the interactions between CNTs and SO_2_ and H_2_S is needed.

O_2_ is an important sensing target. Zettl *et al.*’s reports show that oxygen dramatically affects the physical properties of CNTs [18,19]. However, it is unclear how CNT-based devices respond to O_2_ gas. Avouris *et al.* think that the response of CNT devices to O_2_ is originated from interaction at the SWNT–metal-contact interface which results in a modification of the device SB [20]. It is apparent that considerable future effort will be needed for the development of a usable CNT-based O_2_ sensor.

Organic chemical agents are toxic and very harmful to human health and the environment. These chemicals usually exist in the liquid form, but their vapors can be inhaled and absorbed through the skin. Fast and accurate detection of chemical agents is essential for protecting human health. Novak *et al.* demonstrated that a FET sensor based on a SWNT network can detect dimethyl methylphosphonate (DMMP), a stimulant for the nerve agent Sarin [21]. The sensors were reversible and capable of detecting DMMP at sub-ppb concentration levels (with *V*_GS_ = 0 V). A fast recovery (a few minutes) of the sensor was achieved by applying +3 V gate bias after exposure. The recovery was accelerated by the Coulombic interaction between the negative charge induced by the positive gate bias and DMMP which is a strong electron donor. Similar recovery using back gate bias was observed by Chang *et al.* [22] who found that a negative back gate voltage was required to regenerate SWNT-FET sensors exposed to ammonia and that a positive voltage was required to regenerate sensors exposed to NO_2_. They suggested that this effect could be used to partly circumvent the low sensing specificity of pristine SWNT sensors and allow for a better identification of analytes. Similar FETs were tested with different alcohol vapors with good reversibility and reproducibility by Someya *et al.* [23]. The semiconducting SWNTs were synthesized by CVD and the electrode contacts were formed by evaporating Au with metal shadow masks. When exposed to several saturated vapors of methanol, ethanol, 1-propanol, 2-propanol and *tert*-butanol with *V*_GS_ = −20 V and *V*_DS_ = −100 mV, the response time was within 5–15 s.

Bao *et al.* fabricated thin-film transistor (TFT) sensors consisting of aligned, sorted nanotube networks [24]. This device allows achieving stable low-voltage operation under aqueous conditions. The authors developed a method to enrich semiconducting SWNTs and align them in a one-step solution deposition process by controlling substrate surface chemistry on silicon. These SWNT-TFTs were used to detect trace concentrations, down to 2 ppb, of dimethyl methylphosphonate (DMMP) and trinitrotoluene (TNT) in aqueous solutions. Along with reliable cycling underwater, the TFT sensors fabricated with aligned, sorted nanotube networks enriched with semiconductor SWNTs showed a higher sensitivity to analytes than those fabricated with random, unsorted networks with predominantly metallic charge transport.

Recently, several research groups are working on the functionalization of CNTs with different materials to enhance their sensing performance [25]. There are two main approaches for the surface functionalization of CNTs: covalent functionalization and non-covalent functionalization, depending on the types of linkages of the functional entities onto CNTs. Functionalized CNT sensors often offer a higher sensitivity and a better selectivity, compared with pristine CNT sensors. CNT/polymer nanocomposites as a non-covalent functionalization method without destruction of the physical properties of CNTs offer promising features as a sensing material. An *et al.* [26] fabricated SWNT and polypyrrole (PPy) nanocomposite based gas sensors. The sensor was formed by spin-coating nanocomposites onto prefabricated electrodes. The sensitivity of the nanocomposites was about ten times higher than that of polypyrrole. Abraham *et al.* developed a wireless gas sensor using a multi- walled carbon nanotube (MWNT) and PMMA composite film [27]. The sensor was fabricated by dip-coating the composite film on a pair of electrodes with interdigital fingers. The sensor showed a fast response and a large change of resistivity of an order of magnitude for sensing dichloromethane, acetone, and chloroform.

Challenges for the realization of commercially viable devices are numerous. They include: the development of a detailed fundamental understanding of the sensing mechanisms of these novel sensors and the utilization of this information for the rational design of nanostructured sensing materials; the development of analyte-specific, fast and stable sensors and sensor arrays together with appropriate numerical methods to analyze sensor array data; and the development of suitable high throughput nanomanufacturing techniques that enable mass production of high density sensor arrays.

### 2.3. SWNT-FET Biosensors

Label-free biosensors with high miniaturization and integration have attracted intense research interest since they could potentially make advanced low cost molecular diagnostics routinely available [28–34]. Current sensing options mainly focus on optical detection, using fluorescent-labeled biomolecules with dyes [35–37] or quantum dots [38–40]. The artificial labeling of materials is time-consuming and cost-intensive [41,42], and introduction of labels can weaken the interaction between receptor and target [33]. Hence, the development of fully electronic and label-free sensing techniques is required [28–32]. The electrical conductance of a semiconducting SWNT is sensitive to its environment and varies significantly with surface adsorption of various chemicals and biomolecules [41–43]. This makes SWNT-FETs very promising candidates for label-free biosensing. The SWNT-FETs for biosensor applications are composed of SWNT networks or individual semi-conducting SWNTs. SWNT-FET based biosensors have been reported to detect various biological species such as DNA, proteins and cells.

#### 2.3.1. SWNT-FET Based DNA Sensors

**Figure 2.**
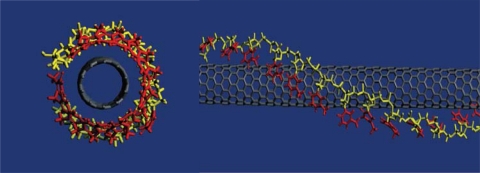
Illustration of a SWNT wrapped by ss-DNA. Aromatic nucleotide bases in the ss-DNA are exposed to form π-stacking with the sidewall of the SWNT; Reproduced with permission from [46].

SWNT-FET sensors which measure DNA hybridization hold great potential for wide scale of genetic testing, clinical diagnostic and fast detection of biological warfare agents. Much attention has been given to the problem of interaction of CNTs and DNA with the purpose for the application in drug delivery and sensing. Nucleic acids including single-stranded DNA (ssDNA) and RNA can disperse SWNTs in water. Interaction of DNA and SWNT in water is due to nucleic acid base π-stacking on the nanotube surface, resulting on the hydrophilic molecular part pointing to the outside. ssDNA has been demonstrated to interact non-covalently with SWNTs [44,45] and form a stable hybrid with individual SWNTs by wrapping around them by means of the aromatic interactions between nucleotide bases and SWNT sidewalls. Molecular modeling suggests that ssDNA can bind to carbon nanotubes through π-stacking, resulting in helical wrapping to the surface (Figure 2). This interaction is dependent on the DNA sequence, which is used for structural separation of SWNTs. Zheng *et al.* demonstrated that bundled SWNTs are effectively dispersed in water by sonication in the presence of ssDNA; these DNA-coated SWNTs can be separated into fractions with different electronic structures by ion-exchange chromatography [46,47]. In addition, theoretical research shows that double-stranded DNA (dsDNA) molecules can interact with SWNTs as major groove binders.

**Figure 3.**
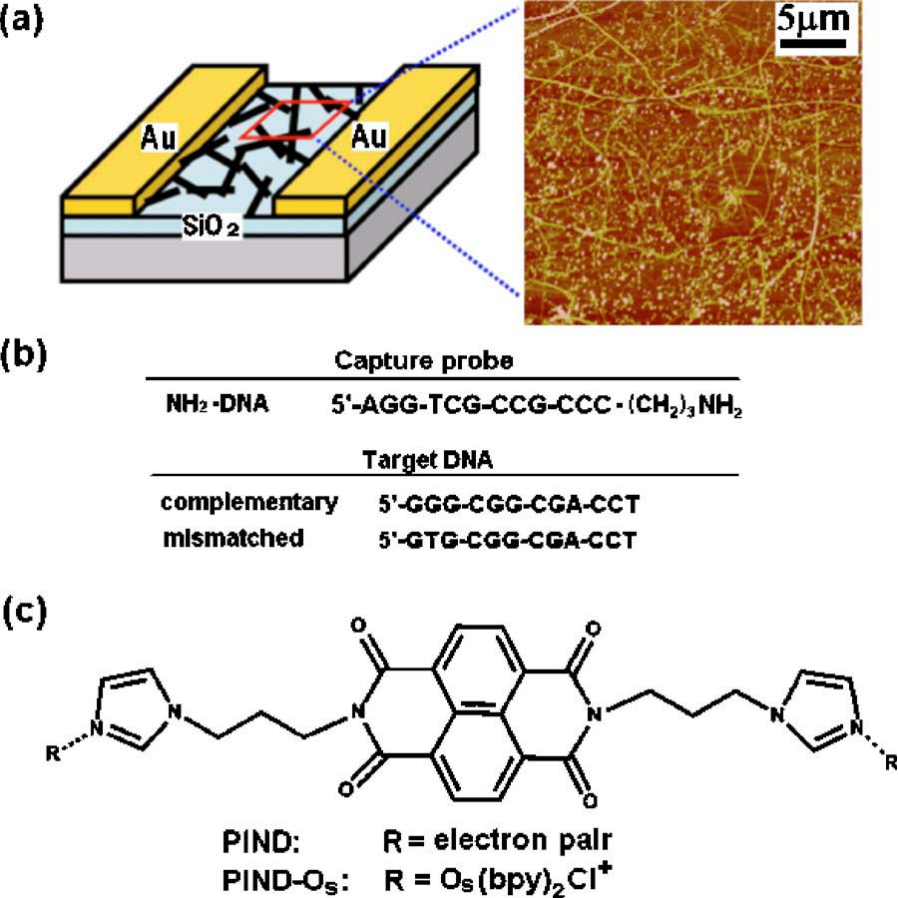
(a) Schematic illustration of the network devices and a typical atomic force microscope image. (b) Sequences of the synthetic oligonucleotide. (c) Structure of PIND and PIND-Os intercalators; Reproduced with permission from [50].

The promising application of functionalized SWNTs in monitoring DNA hybridization was demonstrated by Dekker *et al.* [48]. A coupling peptide nucleic acid (PNA)–an uncharged DNA analogue–was covalently linked to the carboxyl-functionalized tip of SWNTs. Then PNA-DNA hybridization was measured by using atomic force microscopy (AFM). This work laid the foundation for the subsequent development of SWNTs based biosensors. Star *et al.* reported SWNT network FETs that function as selective detectors of DNA immobilization and hybridization. SWNT network FETs with immobilized synthetic oligonucleotides have been shown to specifically recognize target DNA sequences [49]. DNA hybridization with complementary target DNA sequences resulted in reduction of the SWNT-FET conductance. The sensing mechanism relies on the fact that ssDNA adsorbed on sidewalls of SWNTs can result in electron doping to the SWNT semiconductor channels. This SWNT-FET DNA sensor can detect samples with picomolar to micromolar DNA concentrations. Gui *et al.* reported that SWNT-FET immobilized with ssDNA oligomers encoded with a terminal NH_2_ (NH_2_-DNA) show reliable detection and differentiation of its complementary and single-base mismatched DNA strand [50]. The sensitivity was further enhanced by using threading intercalators. Figure 3 shows the schematic illustration of the nanotube network devices and a typical atomic force microscope image showing strands of SWNTs and catalyst particles on them. The device was immobilized with capture DNA probes, and then exposed to complementary or single-base mismatched target oligonucleotides.

**Figure 4.**
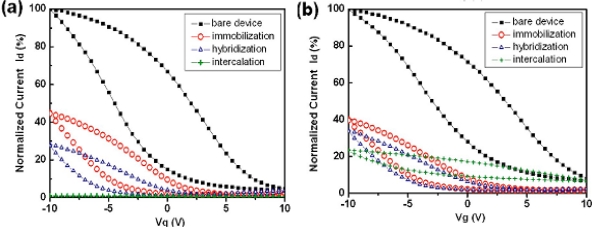
Typical gate voltage dependence of the normalized drain current *I*_DS_ for (a) hybridization with complementary target analyte and (b) hybridization with single-base mismatched target analyte; Reproduced with permission from [50].

The typical transfer curves for SWNT-FET immobilization with NH_2_-DNA was compared with respect to corresponding bare devices (Figure 4a). The SWNT-FET hybridized with the complementary target DNA show a relatively large current reduction as compared to those hybridized with single-base mismatched target DNA The significant reduction in *I*_DS_ for complementary hybridization indicates the formation of a double-stranded DNA which may lead to the increase of scattering centers on semiconductor channels or to shifts in Au work function further away from the valence band of carbon nanotubes. The sensing mechanism of electrical detection of DNA for this network SWNT-FET was further investigated, showing that sensing of DNA is dominated by the variety in metal-SWNT junctions rather than the channel conductance [51]. Maehashi *et al*. reported high sensitive CNT-FET sensor using peptide nucleic acid (PNA) oligonucleotides as the probe, the detection concentration reached as low as 6.8 fM [52].

Dong *et al.* demonstrated the sensitivity of a network SWNT DNA sensor to be enhanced by employing gold nanoparticle (NP) linked DNA target hybrid–DNA detection was observed for concentrations at the femtomolar level [53].

Very recently, Tseng and coworkers have developed a new approach to ensure specific adsorption of DNA to the nanotubes [54]. A polymer was bonded covalently to nanotube in a FET consisting of individual SWNTs. After hybridization, statistically significant changes were observed in key transistor parameters. Hybridized DNA traps both electrons and holes, possibly caused by the charge-trapping nature of the base pairs.

Tao *et al.* reported the unambiguous detection of a sequence of Hepatitis C Virus (HCV) at concentrations down to the fractional picomolar range using SWNT-FET devices functionalized with peptide nucleic acid sequences [55].

#### 2.3.2. SWNT-FET Based Protein Sensors

A great deal of research towards biosensing involves proteins and carbon nanotubes. In this section, some issues including interaction of SWNTs with protein molecules, SWNT-FET protein biosensors, and sensing mechanisms are discussed.

##### 2.3.2.1. Interaction of SWNTs with Proteins

Proteins can strongly bind to the nanotube surface via nonspecific binding (NSB). This NSB interaction is proposed to be in part associated with the amino affinity of CNTs according to recent research. For example, Balavoine *et al.* found that the protein streptavidin binds strongly to the sidewalls of a carbon nanotube in a helical fashion during incubation [56]. Kam and Dai discussed the phenomenon of NSB in a study using carbon nanotubes as protein intercellular transporters [57]. They found that imparting hydrophilicity was insufficient to block this type of binding.

There are many reports that demonstrate the ability to chemically functionalize nanotubes with biomolecules including proteins. Two generalized approaches for this modification of SWNTs are covalent or non-covalent functionalization. As for covalent methods, the SWNTs are usually oxidized to produce free carboxyl groups for coupling with amino groups in proteins. These occur predominantly at tips or defects in SWNTs. The protein is then covalently linked to the carboxyl functionalized SWNTs using standard *N*-hydroxysuccimide/carbodiimide chemistry [58]. In addition, sidewall modification of SWNTs can be performed by using nitrene, cycloaddition, arylation in the presence of a diazonium salt or 1,3-dipolar cycloaddition [59]. Although covalent modification is effective at introducing functionality, it often destroys the desirable mechanical and electrical properties of the SWNTs. On the other hand, the non-covalent approach exploits nondestructive processes and can preserve the primary structures and the unique properties of SWNTs. In term of the non-covalent procedure, certain molecules which act as linkers for biomolecules are first non-covalently coated onto SWNTs, and biomolecules are then covalently bound to the linkers. Dai’s group first demonstrated a two-step hybrid modification of SWNT with proteins [60]: 1-pyrenebutanoic acid succinimidyl ester was first irreversibly adsorbed onto a SWNT via π-stacking interaction; proteins were then immobilized through a nucleophilic substitution of *N*-hydroxysuccinimide by an amino group on the proteins that forms an amide bond. Furthermore, they suggested a strategy to the solve NSB problems [45]. This NSB suppression strategy involves non-covalently coating SWNTs with polyethylene glycol (PEG) containing polymers such as Tween 20. The PEG coating renders the SWNT highly hydrophilic and charge-neutral, thereby eliminating hydrophobic interaction and electrostatic binding with proteins [61]. Star *et al.* demonstrated that the hybrid approach for the immobilization of biomolecule on a PEG/polyethylene imine (PEI) coated SWNT has a high degree of control and specificity [62].

##### 2.3.2.2. SWNT-FET Protein Biosensors

Great effort has been devoted to the development of highly sensitive SWNT-FET protein sensors. The conductance of SWNTs shows sensitive response to a variety of chemical or biological environments. Therefore, biological recognition—such as antibody-antigen interactions—occurring at a SWNT surface can be monitored by electrical measurement of a FET device. To allow for identification of such biological interactions through electrical monitoring, the transfer characteristics of SWNT-FET can be measured using an electrolyte as a top gate or the Si substrate as a back gate. For the back gate transfer characteristics, the current through the drain contact (at fixed drain-source bias) is measured while a variable gate voltage is applied through a back gate buried underneath the SiO_2_ substrate. As far as the electrolyte top gate transfer characteristics are concerned, the device is immersed in a buffer solution and a variable gate voltage is applied through an external electrode immersed in the electrolyte, while the drain current is monitored.

The first demonstration of a CNT-FET protein biosensor was reported by Dekker *et al.* [63]. The device was made of individual SWNTs and a variable electrolyte gate voltage was applied through a platinum electrode. The enzyme glucose oxidase (GO_x_) that catalyzes the oxidation of glucose to glucono-1, 5-lactone, was non-covalently coupled to the sidewall of the SWNTs via a linker molecule of 1-pyrenebutanoic acid succinimidyl ester. The observed decrease in conductance is attributed to the change in the capacitance of the nanotube caused by GO_x_ immobilization. An estimated 50 molecules of GO_x_ were immobilized over the nanotube length of 600 nm. The enzyme layer on the SWNT can inhibit ions in the liquid to come close to the nanotube, thereby decreasing the double layer capacitance and hence the capacitance of the nanotube. However, the possibility of charge scattering at the SWNT in the presence of the GO_x_ molecules cannot be ruled out completely. Dekker *et al.* further demonstrated a strong pH-dependent conductance of the GO_x_ immobilized SWNT, which is presumably due to the pH sensitivity of the charged groups on the GO_x_ molecules, which become more negative with increasing pH. The GO_x_-SWNT system exhibited real time sensing of glucose, thus rendering such a nanoscale FET device as a feasible sensor for monitoring enzymatic activity at single-molecule level. Dai *et al.* reported a general non-covalent approach for the configuration of CNT protein biosensors [45]. Polymers such as Tween 20 or triblock copolymer chains, which are irreversibly adsorbed on nanotubes, act as linkers for binding the biomolecules of interest and as the inhibitor for NSB of proteins. The as-fabricated CNT sensors exhibit selective recognition and binding of target proteins by conjugation of their specific receptors to polyethylene oxide-functionalized nanotubes. In particular, they have studied the affinity binding of 10E3 mAbs antibody (a prototype target of the autoimmune response in patients with systematic lupus erythematosus and mixed connective tissue disease) to human auto antigen U1A.

**Figure 5.**
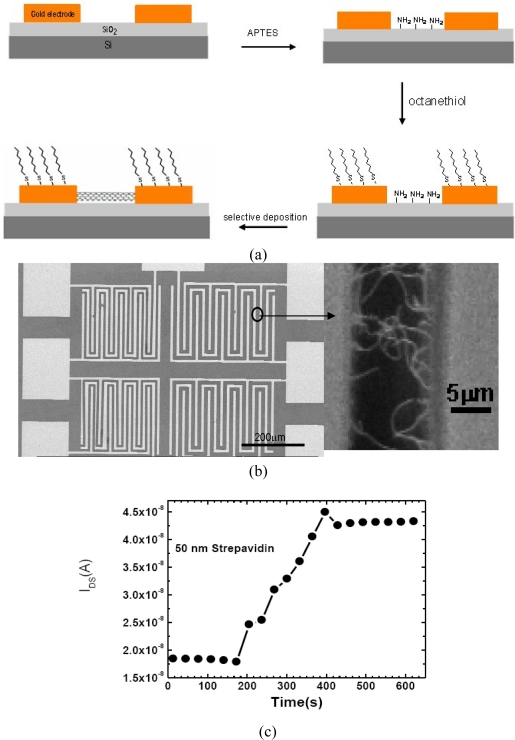

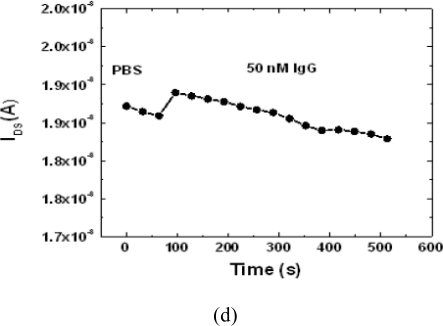
(a) Scheme of the self-assembly procedure of SWNT-FET fabrication. (b) SEM images of the FET chip; magnified images show SWNTs selectively deposited between electrodes, contacting source and drain. The electrode surface is shown to be clean. (c) Real time analysis: time dependence of *I*_DS_ at *V*_DS_ = 0.2 V and at *V*_GS_ = −5 V upon the introduction of target streptavidin (50 nM) onto the biotinylated device. Adding the target streptavidin causes a sharp increase in the source-drain current and then a gradual saturation at slightly lower values. (d) No effect is observed upon the addition of IgG; Reproduced with permission from [61].

Star *et al.* [62] fabricated SWNT-FET biosensors with a back gate, which showed sensitivity to streptavidin by using individual biotin-functionalized SWNTs. The SWNT in the CNT FET device was coated with a mixture of poly ethyleneimine (PEI) and poly (ethylene glycol) (PEG). The PEI provided amino groups for the coupling of biotin-*N*-hydroxysuccinimidyl ester and the PEG prevented the NSB of proteins on the functionalized SWNT. The drain-source current dependence on the gate voltage showed a significant decrease upon streptavidin binding to the biotin-functionalized SWNT. The experiments revealed that specific binding of streptavidin occurred only at the biotinylated interface. The sensing mechanism was explained in terms of the effect of the electron doping of the SWNT channel upon the binding of the charged streptavidin molecules. Hu *et al.* observed a reverse change in the electrical monitoring of biotin-streptavidin interaction using SWNT-FETs [64]. A large area SWNT-FET was fabricated by a self-assembled method whereby the electrode surface and the area between electrodes were modified with nonpolar groups (CH_3_) or polar groups (NH_3_). The SWNTs were selectively placed in the area between the electrodes (Figure 5). The drain-source current at a fixed gate voltage showed an obvious increase upon the binding of streptavidin onto the biotin functionalized SWNT-FET. This increase in conductance upon addition of streptavidin is consistent with binding of negatively charged species to the surface of a p-type SWNT since streptavidin (with an isoelectronic point pI between 5 and 6) is negatively charged at the pH of the measurements (pH 7.2). The sensing mechanism is consistent with electrolyte gating rather than charge transfer as in the work of Star *et al.* Furthermore, Hu *et al.* studied the specificity and real-time detection of the as fabricated CNT-FET biosensor by using bovine immunoglobulin (IgG) as a control protein.

Recently, aptamer modified SWNT FETs have been reported to detect protein. The measurement of antigen-antibody reactions is very common in protein sensing. Aptamers with high affinity and selectivity against a variety of targets such as peptides, proteins and even whole cells can compete with antibodies in biological analysis.

Aptamers have several advantages over traditional antibody-based regents in SWNT-FET biosensors with an electrolyte gate. Firstly, aptamers can be chemically synthesized and be very stable in long term storage, while antibodies are generally produced in organisms. Secondly, the size of aptamers is below the Debye length, which is defined as the typical distance required for screening the surplus charge by the mobile carriers present in a material [65]. If a biomolecule is placed a Debye length away from the surplus charge, its effects on the mobile charges of the material are no longer felt. The typical size of an antibody may vary between 10 and 15 nm [66,67], which is much larger than the Debye length in a typical buffer solution of analytical interest.

Lee *et al.* reported aptamer-CNT FET sensor for biological recognition of thrombin [68]. Maehashi *et al.* demonstrated aptamer-modified CNT-FETs for the detection of immunoglobulin E (IgE) [69]. The SWNTs in the device were covalently immobilized with 5′-amino-modified 45-mer aptamers. The size of the aptamer significantly smaller than the Debye length and that of the corresponding IgE antibody (IgE-mAb). The electrical properties of the SWNT-FETs were monitored in real time. The introduction of target IgE at various concentrations caused a sharp decrease in the source-drain current, and the amount of the net drain-source current before and after IgE introduction on the aptamer-modified CNT-FETs increased as a function of IgE concentration. The detection limit of the as fabricated aptamer modified SWNT-FET was 250 pM. Furthermore, they compared the performance of the aptamer- and antibody-modified SWNT-FET sensors, showing that aptamer-modified SWNT-FETs provided better results than the ones obtained using IgE-mAb-modified SWNT-FETs under similar conditions. Hence aptamer-modified SWNT-FETs are promising candidates for the development of label-free protein biosensors. Recently they developed aptamer-based immunosensors using CNT-FET [70] excepting aptamer, Sim *et al.* reported to exploit another small acceptor (antibody binding fragment) in their SWNT-FET biosensor [71].

#### 2.3.3. Sensing Mechanism of SWNT-FET Biosensors

CNT biosensors have a variety of advantages over conventional chemical and biological sensors due to large surface to volume ratios resulting in higher sensitivity, potential for miniaturization and lower power consumption. In order for CNT sensors to replace conventional sensors, some issues such as the sensing mechanism should be resolved. The physical mechanism underlying sensing is still under debate due to very different conductance effects observed upon sensing, and due to the different interpretations given to similar conductance variations. As previously discussed, Star *et al.* reported a decrease in conductance as a consequence of the biotin-streptavidin interaction on SWNT-FETs and attributed this change to charge transfer, while Hu *et al.* observed a reverse conductance effect from the biotin-streptavidin interaction on SWNT-FET and explained this phenomenon by electrolyte gating. In addition, the observed decrease in transconductance during protein sensing by SWNT-FETs cannot be attributed totally to the scattering of mobile charges. There is the possibility of change in the Schottky barrier (SB) height at the contact of metal-nanotube, which can modulate the conductance of the device. So far, four proposed possible mechanisms that account for the variety in conductance effects of SWNT-FET biosensors are electrostatic gating, capacitance modulation, Schottky barrier effects and carrier mobility change [72–78]. Recently, through modeling and protein adsorption experiments, Dekker *et al.* further studied the mechanisms of biosensing with SWNT-FETs [79]. They discovered that electrostatic gating and Schottky barrier effects are two relevant mechanisms, while electrogating is most reproducible. In particular, the sensing is dominated to be electrostatic gating if the metal electrodes are passivated.

## 3. Graphene-Based Chemical/Biological Sensors

**Figure 6.**
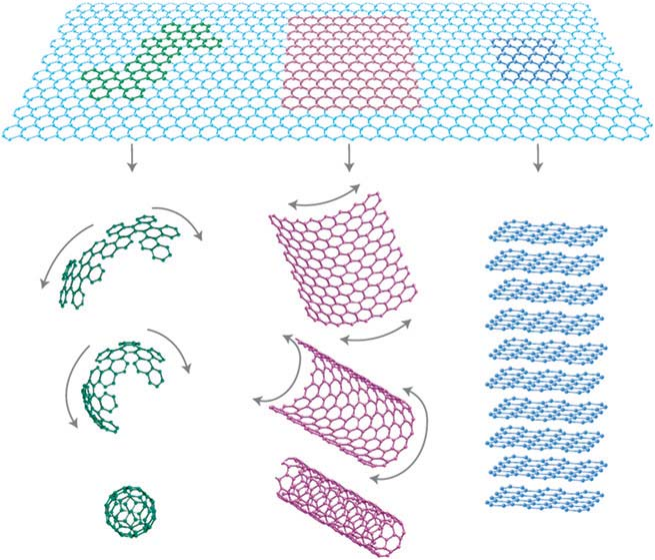
Graphene and its relation to fullerenes, CNTs and graphite; Reproduced with permission from [94].

Following the astonishing discovery of fullerenes and carbon nanotubes in last decades, graphene has recently become an exciting new area in the field of carbon nanoscience and condensed matter physics. Graphene, discovered by Geim *et al.* at the University of Manchester in 2004 [80], is a two dimensional material comprising layers of carbon atoms arranged in six-membered rings. It can be seen as the basis of all carbon materials including fullerenes and carbon nanotubes. Graphene can be wrapped to form fullerene, scrolled to form a carbon nanotube and stacked to form graphite (Figure 6). Graphene was first produced by a micromechanical cleaving technique. This method provided a small amount of high quality samples for fundamental studies. Later on, graphene has also been synthesized by the desorption of Si from SiC single-crystal surfaces [81,82]; by a chemical vapor deposition (CVD) method with a surface precipitation process of carbon in some transition metals such as Ni, Cu, Ru and others [83–88]; and by a chemical solution method [89]. Single-layer graphene has two atoms per unit cell, giving rise to two conical points, K and K_0_ per Brillouin zone, where band crossing occurs, so graphene is a semiconductor with zero bandgap. Graphene exhibits unique properties such as a quantum Hall effect at room temperature [90,91], an ambipolar electric field effect along with ballistic conduction of charge carriers [92], tunable bandgap [93], and high elasticity [94]. In a typical configuration of graphene FET, graphene are deposited on a SiO_2_ layer over a doped silicon substrate; The SiO_2_ layer has a typical thickness of 300 nm, the Si substrate acts as a gate that induces a surface charge density n. This charge density, related to the applied gate voltage Vg through Equation (1) [80]

(1) $$n=\varepsilon_{o}\varepsilon_{d}V_{g}/te≅\alpha V_{g}$$

shifts the Fermi energy level in graphene. In Equation (1), e is the electron charge, ε*_o_* and ε*_d_* are the dielectric permittivities of air and SiO_2_, respectively. These gate-induced carriers are an example of electrical doping, process analogous to the chemical doping typical for semiconductor devices. In contrast to semiconducting devices, where the chemical doping cannot be changed once completed, the chemical p- or n-doping in graphene can be electrically tuned by applying negative or positive voltages, respectively. After the graphene sheet is deposited on the Si/SiO_2_ structure, electrodes can be patterned over it in order to produce certain devices [95].

Graphene is highly promising for the development of new types of chemical/biological sensors with ultrahigh sensitivity due to following reasons: (1) Graphene is a two-dimensional material and thus every atom can be exposed to the surface adsorbates, which maximizes the sensing effect [96]; (2) Graphene’s highly conductive properties and few crystal can ensure a low level of excess (1/*f*) noise caused by thermal switching [97]; (3) The electronic properties of graphene are sensitive to both electron-donor and acceptor molecules, which renders graphene-based devices with high potential for sensing applications.

### 3.1. Graphene-Based Chemical Sensors

**Figure 7.**
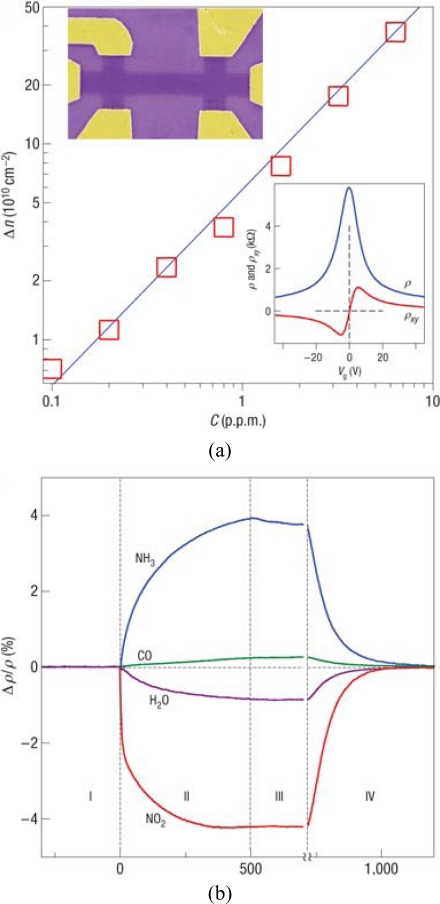

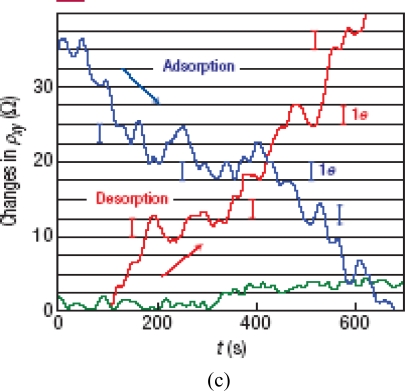
Electrical measurement of graphene gas sensor. (a) Concentration, Δ*n*, of chemically induced charge carriers when the device exposed to different concentrations. (b) Changes in resistivity, ρ with exposure to various 1 ppm gases. (c) Single molecule detection; Reproduced with permission from [98].

Novoselov *et al.* demonstrated a graphene-based sensor with a detection resolution of an individual gas molecule, which is the first reported chemical sensor with the ultimate limit [98]. The graphene devices were prepared by micromechanical cleavage of graphite on an oxidized Si substrate, followed by fabrication of Ti/Au electrodes using electron beam lithography (Figure 7a). To assess the effect of gaseous chemicals on the graphene device, the response to NO_2_, NH_3_, H_2_O and CO with a fixed concentration of 1 ppm was measured. In Figure 7b, region I corresponds to the device before exposure to chemicals. Obvious changes occurred within 1 minute of exposure (Figure 7b, region II) and then the response was followed by a saturation region (Figure 7b, region III). The device can be recovered to the undoped state by annealing in vacuum (region IV). The concentration (Δ*n*) of chemically induced charge carriers as a function of gaseous concentration (*C*) was investigated by measuring the changes in longitudinal (ρ*_xx_*) and Hall (ρ*_xy_*) resistivity. The results showed that Δ*n* depended linearly on the concentration of an examined chemical under the same exposure conditions, which is proposed to simplify the use of graphene-based sensors in practical applications.

To monitor the fundamental limit of graphene gas sensors, a series of techniques were exploited including high driving currents for suppression of the Johnson noise, annealed devices close to the neutrality point, and few-layer graphene devices with low contact resistance. Electrical signals from single-molecule were observed with the above operations (Figure 7c), revealing the adsorption and desorption of individual gas molecule. The chemically induced changes in ρ_xy_ occurred in a step-like manner when the device was exposed to diluted NO_2_. Similar variations on the opposite direction were observed upon stopping NO_2_ supply and starting to evacuate the sample chamber.

Since this first demonstration of a graphene gas sensor, significant research efforts have been made to this field. Recently, Chen *et al.* reported a graphene gas sensor by using partially reduced graphene oxide (GO) [99]. The graphene oxide transistor showed little response to chemical gases such as NO_2_, and the device showed a typical p-type transistor behavior. However, the conductance of GO increased by annealing the device to partially reduce the graphene oxide. The partially reduced GO FET exhibited high sensitivity to exposures to NO_2_ from ∼1.55 to 100 ppm.

Robinson *et al.* demonstrated reduced graphene oxide used for high performance chemical sensors [100]. The sensors are fabricated from a graphene oxide network produced by the Hummers’s method, with the graphene oxides reduced by hydrazine hydrate. The conductance of the graphite oxide device was highly sensitive to the exposure to acetone and some other toxic organics including hydrogen cyanide (HCN), 2, 4-dinitrotoluene (DNT) and dimethyl methylphosphonate (DMMP). The graphene oxide sensors achieved sensitivities at ppb levels for chemical toxics and explosives. The performance of the devices is tunable by varying the exposure time to hydrazine hydrate vapor and the level of reduction affects both the sensitivity and the 1/*f* noise.

### 3.2. Graphene-Based Biosensors

Recently, several reports demonstrated that graphene-based FETs with electrolyte top gating can be efficiently used as chemical/biological sensors. Das *et al.* have discussed monitoring dopants by Raman Scattering in an electrochemically top-gated graphene-based FET [101]. They found that top gating graphene-based FETs was able to reach doping levels of up to 5 × 10^13^ cm^−2^. Such high doping levels are possible because the Debye layer in the electrolyte gate provides much higher gate capacitance. Tao studied the transport properties of graphene FET in ionic solutions, they found that the transport characteristics changes systematically with the ionic concentration, which was qualitatively explained in terms of long-range scattering by charged impurities and ionictheory based on the PB Equation [102]

Modulation of the conductance channel in solution-gated FETs can be achieved by applying a gate potential across the electrolyte, which acts as a dielectric. Because of the ambipolar characteristics of graphene, both hydroxyl (OH^−^) and hydroxonium (H_3_O^+^) ions are proposed to be able to modulate the channel conductance of graphene-based FETs. Loh *et al.* demonstrated solution-gated epitaxial graphene as a pH sensor [103]. The sensor was fabricated from few-layer graphene epitaxially grown on a SiC substrate. The electrochemical double layer at the graphene/electrolyte interface is observed to be very sensitive to pH and the conductance of the device responded to pH changes.

Rao *et al.* studied the noncovalent interaction of DNA nucleobases and nucleosides with graphene by isothermal titration calorimetry [104]. The results showed that the interaction energies of the nucleobases varies in the order guanine (G) > adenine (A) > cytosine (C) > thymine (T) in an aqueous solution. The same trend was found with the nucleosides. Interaction energies of A–T and G–C pairs are somewhere between those of the constituent bases. Theoretical calculations taking into account van der Waals interactions and solvation energies suggest that the trend should be G > A > T > C. Recently, Ohno *et al.* demonstrated an electrolyte-gated graphene-based FET for detecting protein adsorption [105]. The graphene-based FET, which has a non-functionalized single-layer graphene as the channel, exhibited a linear increase in conductance upon the electrolyte pH–thus indicating its potential use in a pH sensor. Further investigation revealed that the conductance of the graphene-based FET increased with the adsorbed protein (bovine serum albumin) at a level of several hundred picomolars, which implied that the graphene-based FET can be used for highly sensitive electrical biosensors. Mohanty and Berry developed a graphene-based single bacterium resolution biodevice and DNA FET [106]. They first investigated the interaction between chemical modified graphene and bioentities. Chemical modified graphene and their biohybrids were synthesized from graphene oxide (GO) or plasma-modified graphene amine (GA). The DNA with a terminal amine group was bonded covalently with carboxylic group of GO by immersing GO coated silica in the amine DNA and amide coupling reagent *O*-(7-azabenzotriazole-1-yl)-*N,N,N,N′*-tetramethyluronium hexafluorophosphate (HATU) solution. This tethering was verified by hybridizing the DNA with a fluorescent cDNA probe. The Gram-positive bacilli cells with highly negatively charged surface were immobilized on GA via electrostatic interactions, which are measured by means of fluorescence microscopy. The electronic conductances of the bacterium device and the DNA transistor were investigated: the results showed that the bacteria biodevice was highly sensitive with a single-bacterium attachment generating ∼1,400 charge carriers in a p-type FET and DNA tethered on graphene hybridizes with its complementary DNA strand to reversibly increase the hole density by 5.61 × 10^12^ cm^−2^. Very recently, Chen *et al.* demonstrated that CVD-grown large-sized graphene films consist-ingmonolayered and few layered graphene domains were used to fabricate liquid gated transistors for DNA sensor with a detection concentration as low as 0.01 nM, they found that adding AuNPs on the surface of graphene devices can extend the upper limit of DNA detectio due to the increase in loading of probe DNA molecules. [107] Leiber *et al*. demonstrated recording from eletrogenic cells using single layer graphene FET as well as simultaneous recording using Gra- and SiNW-FETs. Graphene-FET conductance signals recorded from spontaneously beating embryonic chicken cardiomyocytes which yielded well-defined extracellular signals [108].

## 4. Comparison to other State-of Art Analytic Techniques: Advantages and Graphene/CNT Sensor Challenges

Nowadays, hospital analysis of medically relevant chemicals (gases or vapors) and biomolecules (proteins and DNA) is typically carried out with solid state MOS sensors, spectroscopy, or electrochemical techniques. These techniques require large, expensive and sophisticated equipment. Additionally, present clinical diagnostic techniques exhibit a slow speed since they usually need to treat the sample owing to the low concentration of the analyte of interest. For example, the pre-concentration in the breath–components is employed for diagnostic analysis of medical gas. This procedure limits the diagnostic speed. CNT/Graphene based sensors are proposed to be widely used in medical field in the future since they can provide a cheap, small, fast and high sensitive tool for medical diagnostics.

Since the CNT/Graphene devices render to miniaturization and development of portable sensor. Future carbon nanostructure based sensor is possible to be used for health monitoring or personal diagnostic tool for home use. Another development of CNT/Graphene sensor is applied for monitoring the harmful gas in living environments. Table 1 compares the reported carbon-based sensors and current techniques which used for sensing medically materials such as relevant gases, vapors and biomaterials. Compared to current used medical methods, the carbon nanomaterial based sensor not only have advantagesover. Low energy consumption, potential miniaturization and low cost but also the sensitivity.

## 5. Conclusions

Carbon nanostructures including carbon nanotubes and graphene are central materials for novel sensor architectures due to their structure and excellent electric properties. These carbon nanostructure based FET sensors show a promising future as analysis tools due to their ultrahigh sensitivity and potential for miniaturization.

CNT-based FET sensors have been investigated for nearly ten years, and great progress has been achieved in the field. So far, most of the work has been focused on individual devices. To realize the practical applications of these promising analytic devices, future investigations should emphasize the development of arrays of CNT sensors. Issues concerning production methods, controlled modification of different individual devices with variable sensing materials, and other basic problems need to be resolved.

Research on graphene-based FETs is still in its infancy but these devices show great promise in the fields of electronics and chemical/biological sensors. This new research field offers numerous challenges such as the development of simple reproducible fabrication methods and the understanding of the sensing mechanisms.

## Figures and Tables

**Table 1.** Partial comparison of CNT/graphene sensor to current analytic technique.

**Analyte**	**Current methods**	**Detection limit**	**Carbon material based FET**	**Detection limit**
NO_2_	Spectroscopic	300 ppt	Bare SWNT	100 ppt [11]
			PEI modified SWNT	1 ppb [14]
			Graphene:	single molecule [98]

Organic vapor	GC-MS	ppt to ppb	CNTs	2 ppb [24]
			Graphene	ppb [100]

Protein	Spectroscopic	nM to pM	Aptmer based SWNT-FET	250 p M [69]
			CNTs network	100 pM [45]
			Bare graphene	100 pM [104]
